# Paradoxical Roles of Mineral Dust Induced Gene on Cell Proliferation and Migration/Invasion

**DOI:** 10.1371/journal.pone.0087998

**Published:** 2014-02-04

**Authors:** Miaomiao Yu, Jiaying Sun, Chitra Thakur, Bailing Chen, Yongju Lu, Hongwen Zhao, Fei Chen

**Affiliations:** 1 Department of Pharmaceutical Sciences, Eugene Applebaum College of Pharmacy, Wayne State University, Detroit, Michigan, United States of America; 2 Department of Pulmonary Medicine, Institute of Respiratory Diseases, The First Hospital of China Medical University, Shenyang, China; 3 Liaoning Cancer Hospital and Institute, Shenyang, China; 4 Respiratory Medicine, The 4th Affiliated Hospital, China Medical University, China; University of Pittsburgh Cancer Institute, United States of America

## Abstract

Increased expression of mineral dust-induced gene (mdig, also named as mina53, MINA, or NO52) has been observed in a number of human cancers. The mechanism of how mdig contribute to the pathogenesis of cancer remains to be fully elucidated. In this report, we demonstrated that overexpression of mdig decreased the nuclear staining signal by 4′,6-diamidino-2-phenylindole (DAPI), along with a considerable enhancement in cell proliferation. Silencing mdig by shRNA resulted in a statistically significant decrease of cell proliferation. Intriguingly, mdig overexpression reduced the capacity of the cells in migration and invasion *in vitro*, whereas silencing mdig by shRNA/siRNA enhanced migration and invasion. Clinically, we found that increased expression of mdig in cancer tissues correlates with poorer overall survival of the lung cancer patients, esp., for those without lymph node metastasis. Taken together, our results suggest that mdig plays opposite roles on cell growth and motility, which possibly indicates the paradoxical effect of mdig at the different stages of carcinogenesis.

## Introduction

The basic features of cancer cells include fast proliferation, migration and invasion *in vivo*. Although these features are mutually correlated, each of these features is governed by different biochemical and intracellular signaling pathways. In the earlier stages of carcinogenesis, sustained proliferation is the key for the formation of detectable tumor mass that interferes with the normal functions of the given organs or tissues. During tumor progression, heterogeneous tumor cell subclones arises through genetic and epigenetic evolution of new tumor cell lineages that are able to replenish the cancer cell population and propagate the cells to distant sites [1]. These subclones differ widely in growth, invasiveness, metastatic potential, and their responses to hypoxia condition, chemotherapy drugs and other environmental stressors [2]. Some oncogenic signals act mainly as proliferative factors for tumor cell growth, whereas others may mostly affect the invasiveness or metastasis of the tumor cells.

We had previously identified a mineral dust-induced gene (mdig) in alveolar macrophages isolated from the people with chronic lung diseases resulted from the occupational exposure to mineral dust in mining industry [3], [4]. Mdig was independently identified in c-Myc overexpressing tumor cells and named as myc-induced nuclear antigen 53 (mina53 or MINA) [5], [6], [7], [8]. Since its predominant localization is in the nucleolar compartment of the cells, an alternative name, nucleolar protein 52 (NO52), was used also in literature [9]. A number of human cancers, such as lung cancer [4], colon cancer [10], esophageal squamous cell carcinoma [11], gingival squamous cell carcinoma [12], lymphoma [13], renal cell carcinoma [14], neuroblastoma [15], gastric cancer [16], hepatocellular carcinoma [17], cholangiocarcinoma [18], and breast cancer [19], exhibited increased expression of mdig, indicating important role of mdig played in the pathogenesis of human cancers. How mdig contributes to the development of cancer remains to be fully elucidated.

In the present report, we demonstrated that mdig has different roles in cell proliferation and invasion/migration *in vitro*. Overexpression of mdig in lung cancer cell line, A549, provides the cells with growth advantage. However, at the same time, these cells exhibited reduced potential of invasion and migration.

## Results

### Nuclear, nucleolar and cytoplasmic localization of mdig

**Figure 1:**
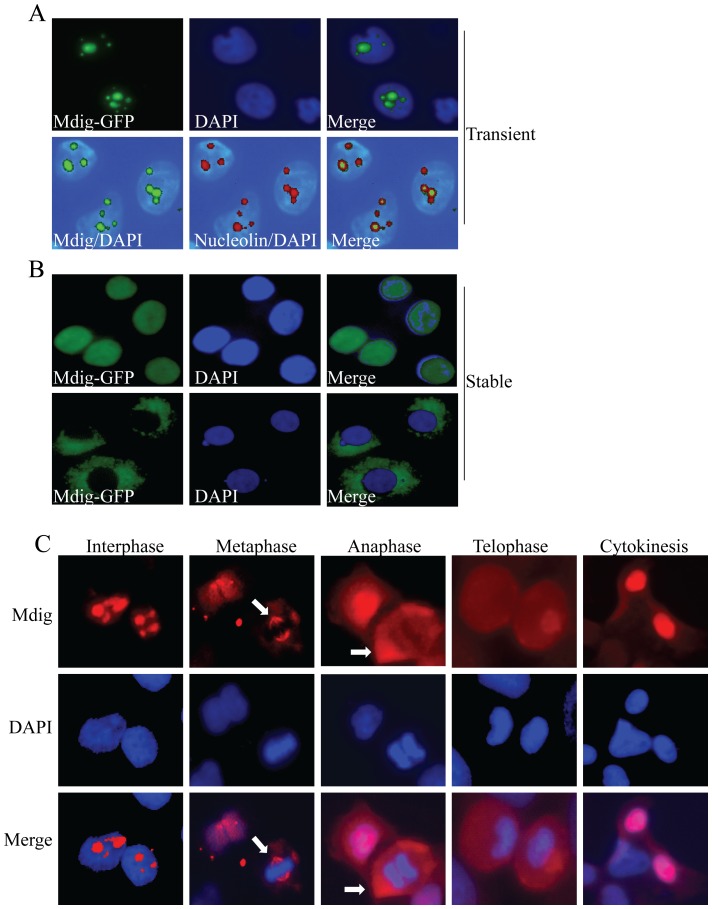
Mdig is localized in nucleolus, nucleus and cytoplasm. A. Immunofluorescent staining shows nucleolar localization of the transiently transfected mdig-GFP (green, upper panels). Lower panels show co-localization of mdig with the nucleolar protein, nucleolin (red). B. Some of the stably transfected cells show localization of mdig in the nuclear matrix and cytoplasm. C. Endogenous mdig is located in nucleoli in interphase and in the mitotic spindle (pointed by white arrows) during metaphase and anaphase of the cell cycle.

Earlier studies suggested that mdig/mina53/NO52 is predominantly localized in the nucleolar compartment of the cells [4], [5], [9]. To investigate whether the intracellular distribution of mdig may be altered under certain circumstances of the cell growth conditions, we evaluated mdig localization in detail through immunofluorescent microscopy. In transient transfection of the A549 lung cancer cells with mdig-GFP expression vector, we noted that majority of the cells showed nucleolar localization of the exogenous mdig protein (Fig. 1A). The nucleolar localization was verified by the co-localization of the mdig protein with nucleolin, a protein exclusively located in nucleoli of the cells (panels in the 2^nd^ row, Fig. 1A). In stable mdig transfected cell lines, however, we observed that mdig was located in the entire nuclear matrix in some cells (Fig. 1B). In one of the several stably transfected clones, we even noted that mdig is localized in the cytoplasmic compartment of the cells exclusively (Fig. 1B, bottom panels).

The intracellular localization of the endogenous mdig was also determined for the cells at different cell cycle phases. In interphase cells (G0/G1 phase, S phases, and G2 phases), the endogenous mdig was mainly found in the nucleoli of the cells (Fig. 1C, the most left column). When the cells entered into the metaphase and anaphase of the mitotic phase (M phase), a co-localization of mdig with the mitotic spindle structure was observed (Fig. 1C, pointed by white arrows). In both telophase and cytokinesis, mdig was evenly distributed in the nuclear matrix.

### Altered intracellular localization of mdig in response to cellular stress

**Figure 2:**
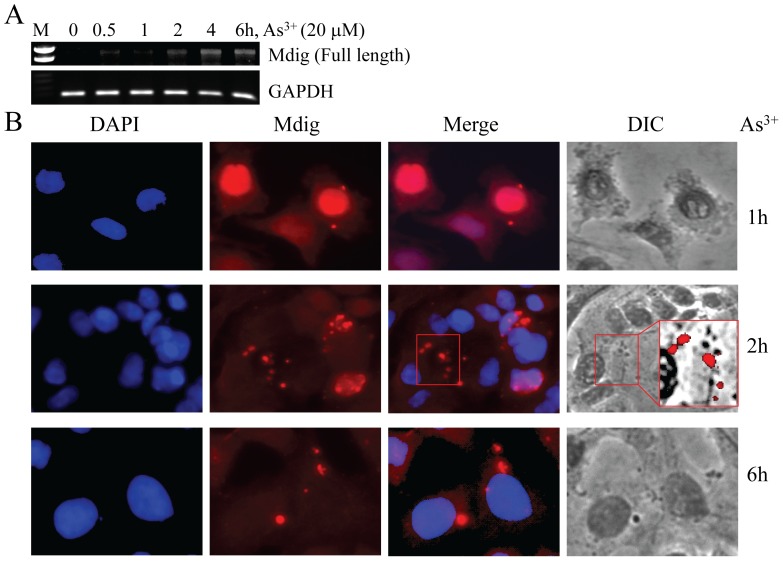
Arsenic (As^3+^) increases mdig expression and cytoplasmic localization. A. As^3+^ induced mdig mRNA in A549 cells as detected by RT-PCR. B. Immunofluorescent staining shows cytoplasmic localization of mdig in response to As^3+^ treatment. The fields showing cytoplasmic localization of mdig in Merge panel and stress granules in Differential Interference Contrast (DIC) panel (framed by red boxes) were merged together and magnified as an insert in DIC panel.

As we reported before, some extracellular stimuli can induce expression of the mdig gene [3], [4]. To determine whether these stimuli alters intracellular localization of the mdig protein also, we treated the A549 cells with 20 µM sodium arsenite (As^3+^), a typical stress inducer, for 0.5 to 6 hours (h) followed by RT-PCR to measure the levels of mdig mRNA and immunofluorescent staining for the determination of intracellular distribution of the mdig protein in these cells. As depicted in Fig. 2A, As^3+^ was able to induce the accumulation of mdig mRNA that was peaked at 4 h and remained high at 6 h. Immunostaining of mdig revealed that at 1 h of As^3+^ treatment, mdig was mostly localized in nuclei. After 2 to 6 h treatment, some cells showed a punctuation or speckled pattern of the mdig localization in the cytoplasm. These punctuated mdig staining signals were not uniform in size. Although As^3+^ treatment appears to be able to increase the number of stress granules, most of the punctuated mdig staining signals were not overlapping with these granules (insert framed by red line box in Fig. 2B), suggesting that these cytoplasmic mdig speckles are not within the stress granules of the cells.

### Mdig enhances cell proliferation

**Figure 3:**
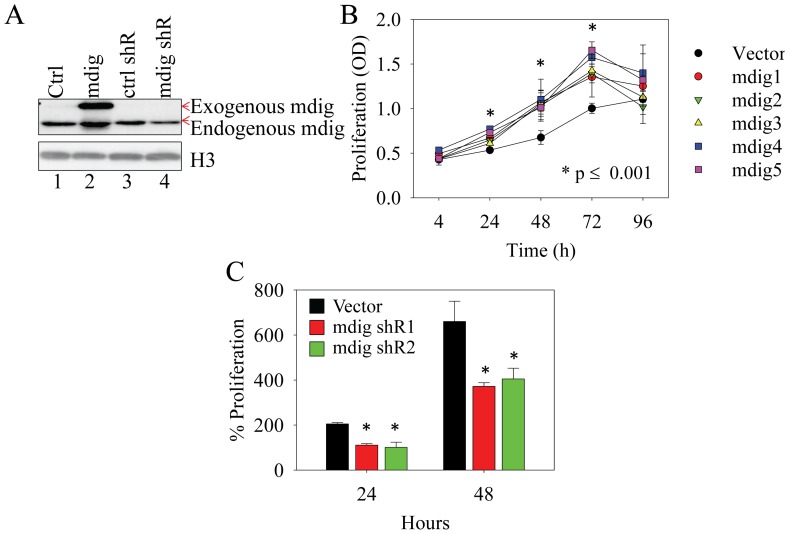
Mdig is a pro-proliferative factor for cell growth. A. Westernblotting shows protein levels of exogenous and endogenous mdig (pointed by red arrows) in the cells stably transfected with mdig-GFP or shRNA that specifically silences mdig expression. B. MTT-based cell proliferation assay indicates that mdig overexpression enhances cell proliferation. Five independent stably mdig-transfected clones were analyzed. C. Silencing mdig by two individual shRNAs that target different region of the mdig mRNA suppressed cell proliferation. Ctrl: control; shR: shRNA.

We had shown that mdig promotes cell growth of the immortalized but non-tumorigenic human bronchial epithelial cell line, BEAS-2B, possibly through regulating cell cycle transition from G1 phase to S phase [3]. To determine whether this effect can be seen in the tumor cell line, we assessed cell proliferation rate of the A549 cells following stable transfection of mdig expression vector or vectors containing mdig-targeting shRNA. The expression of exogenous mdig and the silencing effect of mdig shRNAs on mdig were validated by Westernblotting experiments (Fig. 3A). As depicted in Fig. 3B, all 5 mdig stable expression clones showed an enhanced cell proliferation at 24, 48 and 72 h of culture. At 96 h, no statistically significant difference was observed in cell growth between the mdig expressing cell clones and the cells transfected with a control vector, possibly due to saturation of the cell density in culture and the exhaustion of nutrients in the culture medium.

The pro-proliferative role of mdig was further confirmed in the cells stably expressing shRNAs that silence mdig. At both 24 and 48 h of cell culture, a significantly reduced proliferation was noted in the cells expressing mdig shRNA relative to the cells expressing a control shRNA (Fig. 3C).

### Mdig suppresses cell migration and invasion

**Figure 4:**
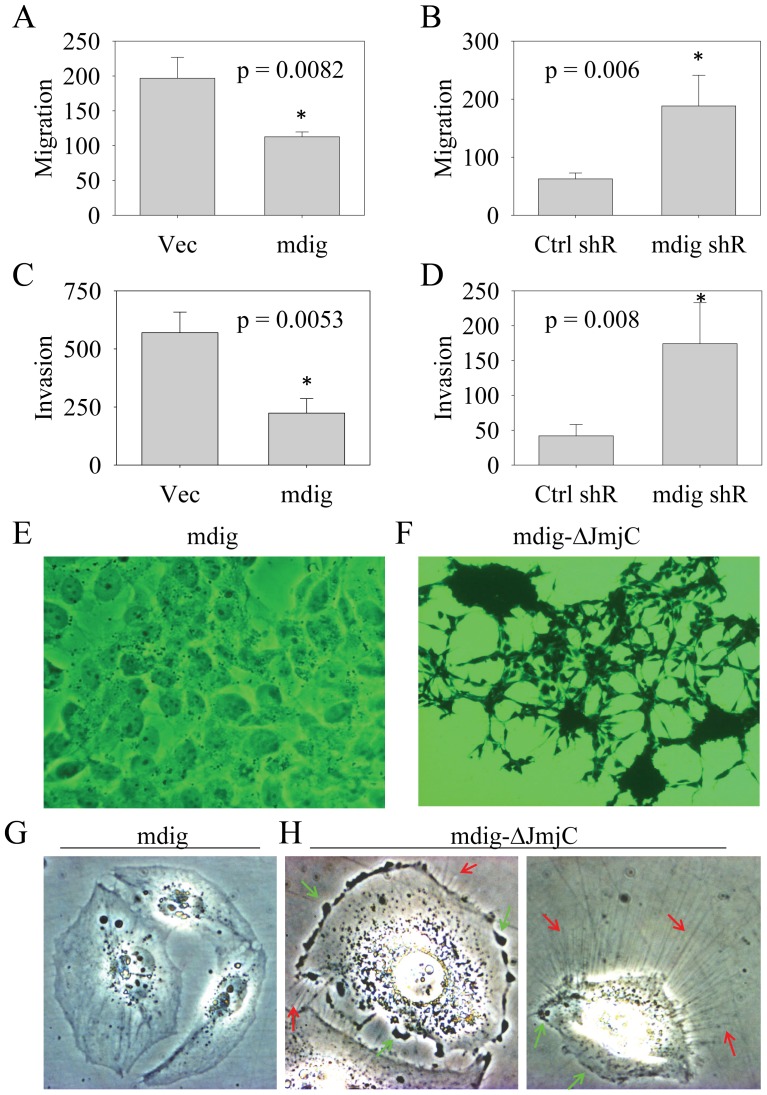
Mdig inhibits cell migration and invasion. A & B. Migration assays show decreased and increased migration of the cells stably expressing mdig (A) and the mdig specific shRNA (B), respectively. C & D. Invasion assays show decreased and increased invasion of the cells stably expressing mdig (C) and the mdig specific shRNA (D), respectively. E–H. Inhibition of mdig by overexpressing a JmjC domain-deletion construct of mdig alters cell morphology of the BEAS-2B cells.

Prompted by the proliferative role of mdig on cell growth, we'd like to know if mdig is involved in the regulation of cell migration and invasion, general features of the malignant cells. By using a modified Boyden chamber assay, the capabilities of the cells in migration and invasion *in vitro* were determined. In migration assay, the inserted filter was uncoated. For invasion assay, the filter membrane of the inserters was pre-coated with Matrigel. It is unexpected that mdig-expressing cells showed reduced abilities in migration and invasion. A 40–45% reduction in migration and a 50–60% reduction in invasion were observed in the mdig-overexpressing cells relative to the cells expressing a control vector (Figs. 4A & 4C). To verify the suppressive role of mdig on cell migration and invasion, we silenced mdig expression by shRNA to determine whether the impaired capabilities of the cells in migration and invasion could be restored. As depicted in Figs. 4B & 4D, a significant increase in either migration or invasion was detected among the cells in which mdig expression was silenced by shRNA. These data, therefore, suggest that mdig is an inhibitory factor for cell migration and invasion.

The repressive role of mdig on cell migration and invasion was unexpected but might explain the observed morphological changes of the human bronchial epithelial cells (BEAS-2B) stably expressing a deletion-mutated mdig in our previous studies [4]. In BEAS-2B cells, overexpressing a deleted construct of mdig by removing the conserved JmjC domain repressed cell growth and altered the cuboidal-like shape of the cells into a thin and elongated fibroblast-like morphology (Fig. 4F). Moreover, compared to the smooth edge of the vector or mdig-transfected cells (Fig. 4G), the cells expressing the JmjC-deleted mdig showed an increased formation of membrane ruffles (pointed by green arrows) and protrusions (pointed by red arrows), in addition to swollen and poorly defined organelles in the cytoplasm, indicative of active migration of the cells (Fig. 4H). It is very likely that mdig may indirectly retain the GTPase activities of Cdc42 or Rac1 that alter G-/F-actin ratio and promote filopodium formation to enhance cell migration. Inhibition of mdig by either shRNA or expressing the JmjC-deleted mdig, thus, may release such retention and foster cell migration and invasion.

### Mdig is a prognostic factor for the survival of the lung cancer patients

**Figure 5:**
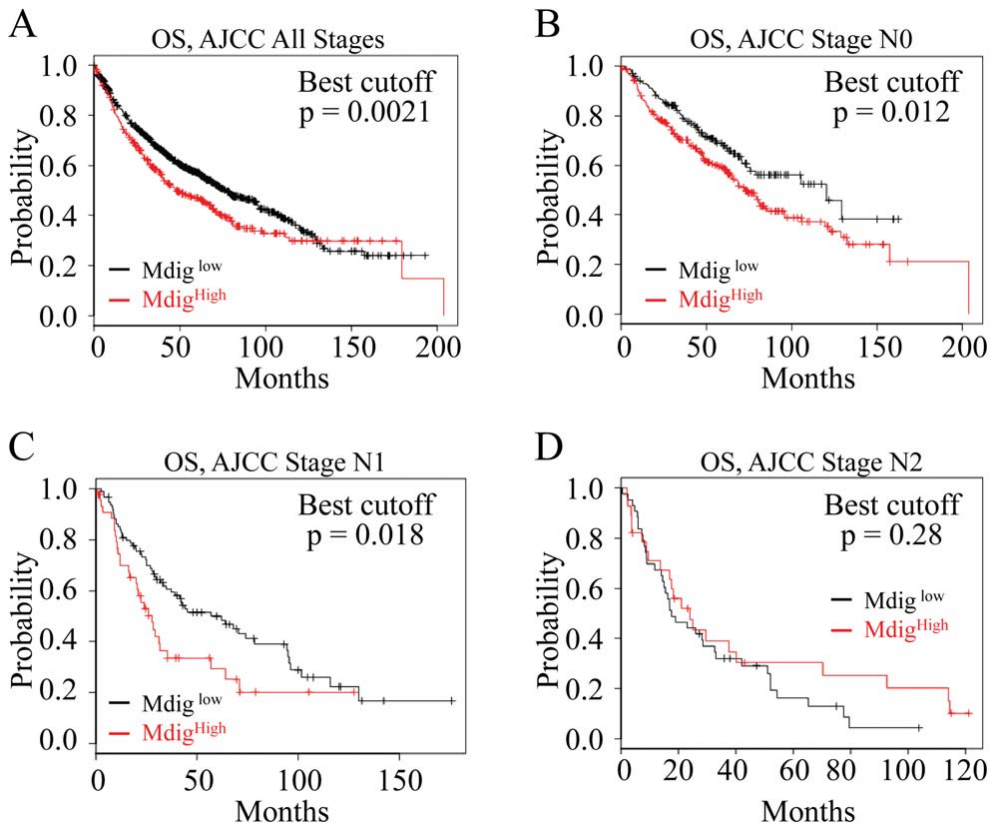
Increased mdig expression predicts poorer overall survival (OS) of the lung cancer patients. A. Higher level of mdig predicts poorer OS of the total lung cancer patients. B. Higher level of mdig predicts poorer OS of the lung cancer patients without lymph node metastasis (American Joint Committee on Cancer N0, AJCC N0). C. Higher level of mdig predicts poorer OS of the lung cancer patients with possible proximal lymph node metastasis (AJCC N1). D. Higher level of mdig can not predict poorer OS of the lung cancer patients with distal lymph node metastasis (AJCC N2).

To explore whether the above findings are clinically relevant for the lung cancer patients, we analyzed mdig expression level and the survival data of 1,715 lung cancer patients using an online gene profiling database by stratifying patients based on the higher or lower mdig expression [20]. We found that mdig levels are inversely correlated with the overall survival (OS) of the lung cancer patients. Higher expression of mdig predicts poorer OS of the lung cancer patients with a statistically significant p value smaller than 0.005 (p = 0.0021, Fig. 5A). This observation may compensatory support the finding indicating pro-proliferative role of mdig overexpression in both BEAS-2B cells we reported before [3] and the A549 cells as reported here (Fig. 3). However, if we classified the patients based on the American Joint Committee on Cancer (AJCC) staging of lymph node (N) metastasis status, we noted higher mdig expression only predicted poorer OS of AJCC N0 (no regional lymph node metastasis) and AJCC N1 (possible proximal lymph node metastasis) patients, but not the AJCC N2 patients who had distant lymph node metastasis (Figs. 5B, 5C and 5D). Although statistically insignificant, higher mdig expression appears to be able to predict better, rather than poorer, survival of the AJCC N2 patients (Fig. 5D), which may support the findings that mdig is inhibitory for cell migration and invasion (Fig. 4).

## Discussion

A number of human cancers exhibited increased expression of mdig gene, including lung cancer [3], [4], colon cancer [13], and breast cancer [19], which implies important contribution of mdig to the pathogenesis of human cancers. Earlier studies had shown a pro-proliferative role of mdig for some established human malignant or non-malignant cell lines and consequently linked mdig gene as one of the newly defined oncogenic genes. Despite studies that revealed mdig as a possible regulator for cell cycle transition, it is unclear whether mdig affects other aspects of the cancerous cells, such as cell motility and invasiveness. In the present report, we provide evidence showing that mdig protein may localize in the nuclear matrix and cytoplasm, in addition to nucleolar compartment of the cells under overexpression or stress conditions. The overexpression of mdig promotes cell proliferation of the A549 lung cancer cells, which confirmed previous findings in other types of cells. It is unexpected, however, that mdig overexpression represses cell migration and invasion of the A549 cells, suggesting paradoxical role of mdig in cell growth and motility.

The opposite effects of mdig on cancer cell proliferation and motility or invasion may be clinically relevant for the pathogenesis of the lung cancer and the prognosis of the lung cancer patients. Analyses of the mdig expression levels and the patient survival data clearly suggest that increased expression of mdig predicts poorer overall survival (OS) of the total lung cancer patients (Fig. 5A) and the poorer OS of the lung cancer patients without lymph node metastasis or only having possible proximal lymph node metastasis (Figs. 5B and 5C). In contrast, such a prediction of poorer OS was not observed for the lung cancer patients who had distal lymph node metastasis (Fig. 5D). In fact, increased mdig expression appears to be able to predict better OS of the patients who had distal lymph node metastasis, although it was statistically insignificant. These observations are in agreement with what we observed of the prognostic value of mdig for the breast cancer patients [19]. In breast cancer, increased mdig predicts poorer OS of the patients who have no lymph node metastasis but better OS of the patients who have signs of lymph node metastasis.

By studying mdig/mina53 expression in lung cancer tissues from 101 patients and correlating the mdig expression with the patient survival time, Komiya et al. [21] reported that mdig/mina53 overexpression is associated with favorable prognosis of the lung cancer patients, esp., for the stage I lung cancer patients. Such a favorable outcome was not observed in AJCC N0 and AJCC N1 patients in our studies. Instead, increased mdig expression clearly predicted poorer OS of these AJCC N0 and N1 patients. A possible favorable prognosis of mdig overexpression may present for the AJCC N2 patients. The discrepancy between Komiya et al. and the present report may be resulted from the differences in sample sizes and ethnic groups of the patient population studied.

Since its first discovery from human lung alveolar macrophages [3] and tumor cell lines [5], the detailed mechanisms of mdig in mediating cell growth and carcinogenesis remain to be fully elucidated. Based on the conserved JmjC domain in mdig protein, several attempts had been made to determine whether mdig/mina53 has the demethylase activity. Although our previous and current studies showed some alterations in the trimethylation of lysine 9 of histone H3 (H3K9me3), inconsistence occurred in the different experimental settings. For example, either overexpression of the exogenous mdig or silencing the endogenous mdig by siRNA/shRNA in lung epithelial cells or lung cancer cells showed very marginal changes on the global levels of H3K9me3. However, increased mdig expression was able to reduce H3K9me3 in the regulatory regions of Oct4 and the large intergenic non-coding RNA (lincRNA) H19 gene, whereas silencing mdig increased the abundance of H3K9me3 in the regulatory regions of Oct4 and H19 [22]. This raises the question whether mdig/mina53/NO52 has some site-specific effect on the levels of H3K9me3 among a limited number of genes. An additional question remaining to be answered is whether mdig is a co-factor of other histone demethylases or other co-factors are needed for the activity of mdig. Lastly, it will be interesting to know whether the observed antagonistic role of mdig on migration and invasion in the present study is resulted from effects of mdig on cytoskeleton structures, cell adhesion signaling, and/or enzymes that degrade extracellular metrix. Answering these questions will shed new light on our understanding of how mdig contributes to the pathogenesis of human cancer and the prognosis of the cancer patients.

## Materials and Methods

### Cell culture and transfection

Human lung adenocarcinoma epithelial cell line A549 and human bronchial epithelial cell line BEAS-2B are purchased from the American Type Culture Collection (ATCC, Manassas, VA). The cells were maintained in RPMI1640 or DMEM medium (Invitrogen, Grand Island, NY) supplemented with 5% fetal bovine serum and 1% penicillin-streptomycin (Sigma, St. Louis, MO) in 37°C humidified incubator in the presence of 5% CO_2_. For cell transfection, 5×10^5^ cells were seeded in 6-well plates. Cell transfection was performed when the cells reached to 60–70% confluence using Lipofectamine 2000 (Invitrogen). The mdig-GFP expression vector, control vector, RFP-conjugated mdig shRNAs, and RFP-conjugated control shRNA were purchased from OriGene (Rockville, MD). For establishing stably transfected clones, the cells were cultured in the media containing 2 µg/ml of puromycin for 3 weeks. The GFP- or RFP-positive clones were maintained for subsequent analyses of cell proliferation, migration, invasion, and additional immunofluorescent staining or Western blotting.

### Western blotting

Total cellular proteins were prepared by lysing the cells in RIPA buffer (Millipore, Billerica, MA) supplemented with phosphatase/protease inhibitor cocktail and 1 mM PMSF through sonication and centrifugation, followed by quantification using a Micro BCA Protein Assay Reagent Kit (Thermo Scientific, Pittsburgh, PA). Before loading on 10% SDS-PAGE gels, the proteins were boiled in LDS sample buffer (Invitrogen) containing 1 mM dithiothreitol. The separated proteins were transferred onto PVDF membranes (Invitrogen) and subjected to immunoblotting with the indicated antibodies.

### RT-PCR

Total RNAs were extracted from the cells using TRIzol Reagent and subjected to RT-PCR using AccessQuick RT-PCR system purchased from Promega (Madison, WI). The PCR primers for mdig are: 5′-TCA TGT CGG GCC TAA GAG AC-3′ and 5′-GGC ATT TGA TTC TGC AAA GG-3′, which amplifies a 1,510 bp DNA fragment covering the whole coding region of the mdig gene. PCR primers for GAPDH are: 5′-CTG AAC GGG AAG CTC ACT GGC ATG GCC TTC-3′ and 5′-CAT GAG GTC CAC CAC CCT GTT GCT GTA GCC-3′.

### Cell proliferation assay

Cell proliferation rate was determined by seeding 5×10^3^/per well in 500 µl medium in 96-well plates. At the indicated time points after seeding, 20 µl of MTT solution (5 mg/ml in PBS, Sigma) was added to each well and incubated at 37°C for another 3.5 h. At the end of incubation, the medium was replaced with 150 µl of DMSO (Fisher) to dissolve the formazan crystals. After agitation on shaker for 15 min, absorbance at 570 nm–690 nm was measured using a Biokinetics plate reader (Bio-Tek Instruments, Inc, Winooski, VT, USA)

### Migration and Invasion assay

Cell migration and invasion were determined using BD BioCoat™ Matrigel™ Invasion and Migration Chambers according to the manufacturer's instruction. After incubating for 24 h (migration) or 48 h (invasion), the cells in the upper chambers were scrubbed out using cotton tipped swab. The cells on the lower surface of the membrane were stained with the Diff-Quik Kit. The migrated and invasive cells were counted under a microscope.

### Survival analysis and statistics

A Kaplan-Meier survival database that contains survival information of 1,715 lung cancer patients and gene expression data obtained by using three different versions of Affymetrix HG-U133 microarrays was used [23]. Two different mdig probe sets, probes 213188_s_at and 213189_at, are presented in this database. Probe set 213188_s_at was excluded because it detects the far end of the 3′-UTR of mdig mRNA and the antisense of 3′-UTR of the β-γ-crystallin domain containing 3 (CRYBG) mRNA. The probe set 213189_at that detects the open-reading frame (ORF) of mdig mRNA was used in this survival analysis. Survival curves resulting in *p* values of <0.05 between mdig higher (mdig^high^) and mdig lower (mdig^low^) groups were considered significantly different. The best performing threshold (best cutoff) was used as a cutoff of the survival data.
